# Cellular heterogeneity in DNA alkylation repair increases population genetic plasticity

**DOI:** 10.1093/nar/gkab1143

**Published:** 2021-11-25

**Authors:** Maxence S Vincent, Stephan Uphoff

**Affiliations:** Department of Biochemistry, University of Oxford, South Parks Road, Oxford, OX1 3QU, UK; Department of Biochemistry, University of Oxford, South Parks Road, Oxford, OX1 3QU, UK

## Abstract

DNA repair mechanisms fulfil a dual role, as they are essential for cell survival and genome maintenance. Here, we studied how cells regulate the interplay between DNA repair and mutation. We focused on the adaptive response that increases the resistance of *Escherichia coli* cells to DNA alkylation damage. Combination of single-molecule imaging and microfluidic-based single-cell microscopy showed that noise in the gene activation timing of the master regulator Ada is accurately propagated to generate a distinct subpopulation of cells in which all proteins of the adaptive response are essentially absent. Whereas genetic deletion of these proteins causes extreme sensitivity to alkylation stress, a temporary lack of expression is tolerated and increases genetic plasticity of the whole population. We demonstrated this by monitoring the dynamics of nascent DNA mismatches during alkylation stress as well as the frequency of fixed mutations that are generated by the distinct subpopulations of the adaptive response. We propose that stochastic modulation of DNA repair capacity by the adaptive response creates a viable hypermutable subpopulation of cells that acts as a source of genetic diversity in a clonal population.

## INTRODUCTION

Genome plasticity is essential for adaptation of cells to new environments. For instance, bacteria rely on mutagenesis to evolve resistance to antibiotics (1–3) and to adapt to new host environments (4). On the other hand, maintenance of genome stability is also necessary for their survival. Hence, cells employ conserved genetic networks and stress responses to regulate repair of their DNA (5). Perturbation of DNA repair pathways by mutations or drug treatments increases the mortality and mutation rates of cells in the presence of DNA damage. Loss of repair functionality can have beneficial consequences for bacterial populations, as an increased mutation rate can enhance evolvability. Indeed, mutator strains consistently evolve during laboratory evolution experiments (6,7) and are frequently found in bacterial isolates from infected patients or the environment (8,9). These phenotypes have been shown to arise from mutations in DNA mismatch repair, oxidative DNA damage repair and DNA replication proofreading genes. However, although an increased mutation supply can accelerate adaptive evolution when a population is maladapted in its current environment, inactivation of genome maintenance mechanisms can lower cell fitness and lead to accumulation of deleterious mutations (10). Besides the existence of permanent genetic mutator alleles, growing evidence suggests that cells can adopt transient hypermutable phenotypes by regulating the expression or activity of DNA repair enzymes (11–14). Temporary upregulation of mutagenesis is believed to promote evolutionary adaptation in response to stress without compromising genetic stability in optimal environments (15,16). Furthermore, cell subpopulations with elevated mutation rates could serve as reservoirs of increased genetic plasticity. Despite the compelling logic of this theory, whether a hypermutable subpopulation contributes significantly to the overall evolvability of the whole population depends not only on its mutation rate but also on its size, lifetime and viability (17). These crucial parameters are not accessible from conventional genetics assays. As such, it remains unclear if transient hypermutable phenotypes can provide evolutionary benefits, and how any such benefits compare to the evolvability of permanent genetic mutator strains.

**Figure 1.**
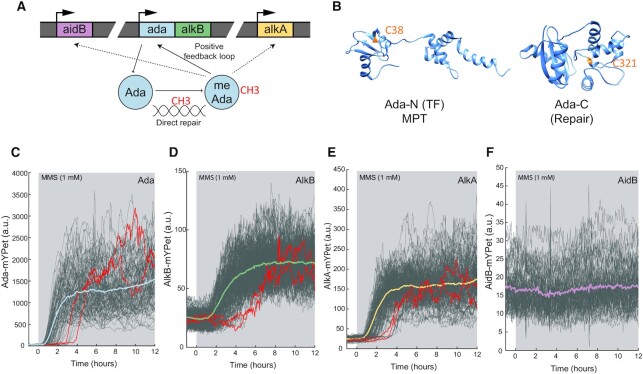
Stochastic induction of the adaptive response genes in response to alkylation stress. (**A**) Schematic of the adaptive response regulation. The adaptive response gene network is composed of the *ada-alkB* operon, *alkA* and *aidB*. Methylation of the damage sensor protein Ada turns itself into a transcriptional activator the regulon. (**B**) Ada N-terminal domain (PDB: 1ZGW) and C-terminal domain (PDB: 1SFE) carry the methylated phosphotriester (MPT) and O^6^meG repair activities, respectively. The methyl acceptors C38 and C321 are shown in orange. (**C–F**) Microfluidic-based imaging of the expression levels of adaptive response proteins upon 1 mM MMS treatment (shaded background). Single-cell time-traces of the average fluorescence intensity per cell for Ada-mYPet (cells = 104) (**C**), AlkB-mYPet (cells = 265) (**D**), AlkA-mYPet (cells = 228) (**E**) and AidB-mYPet (cells = 146) (**F**). Example of cells with delayed gene induction are shown in red. Coloured curves represent the cell average fluorescence intensity time trace.

Among the broad class of damaging compounds that can generate mutagenic DNA lesions, alkylating agents are found in the external environment (18) and are endogenously produced (19,20). They can alter nucleobases and phosphotriester linkages of single- and double-stranded DNA, and RNA in eukaryotic and prokaryotic cells (21–24). In *Escherichia coli*, six genes have been identified that protect DNA specifically against alkylation damage. The two constitutively expressed enzymes, Ogt (O^6^meG methyltransferase) and Tag (3meA DNA glycosylase I), provide a basal repair capacity (25–28), whereas the four proteins of the adaptive response, Ada (O^6^meG methyltransferase), AlkA (3meA DNA glycosylase II), AlkB (3meC dioxygenase) and AidB, are induced upon alkylation stress (29–34). *ada* and *alkB* are expressed in an operon, while *alkA* and *aidB* have separate promoters (Figure 1A).

The adaptive response is regulated through the methylation status of Ada (Figure 1A). Ada is a bifunctional enzyme, exhibiting a transcription factor activity carried by its N-terminal domain (Ada-N) and an O^6^meG methyltransferase activity with the catalytic cysteine 321 (C321) in the C-terminal domain (Ada-C) (Figure 1B). Ada-N repairs methylated phosphotriester lesions by direct and irreversible transfer of the methyl group onto its catalytic cysteine 38 (C38). The methylation of C38 turns Ada into a transcriptional activator of the adaptive response gene network, which includes its own gene and thus leads to amplification of gene expression by positive feedback (18,35,36).

Although the adaptive response has been characterized for decades, recent single-cell measurements uncovered unexpected cell-to-cell heterogeneity in Ada abundance (37). Specifically, *ada* exhibits large variation in gene expression between cells of isogenic *E. coli* populations (37). As a result of gene expression noise, the basal level of Ada in absence of alkylation stress is heterogeneous, with a subpopulation of cells containing not even a single molecule of Ada. Consequently, upon alkylation stress, cells devoid of Ada are unable to activate the adaptive response until the stochastic expression of at least one Ada molecule, which can take multiple cell generations (37,38). Cells with a delayed adaptive response exhibit higher rates of DNA replication errors, suggesting that they could act as a hypermutable subpopulation (37,39). However, as phenotypic variation is ubiquitous in bacteria, it is difficult to know whether the heterogeneity in the adaptive response has a functional outcome or if it is a side-effect of unavoidable molecular noise. Here, we addressed this question by studying the regulation of the adaptive response and its effects on population genetic plasticity.

## MATERIALS AND METHODS

### Construction of strains and plasmids

All strains were derived from *Escherichia coli* K12 AB1157. C-terminal msCFP3, mYPet and HaloTag fusions were inserted with a flexible 11 amino acids linker (SAGSAAGSGEF) at the endogenous chromosomal loci by λ-red recombination using plasmids pSU003 (37), pRod50 (40) and pSU005 (41). The λ-red insertions are flanked by Flp site-specific recombination sites (frt) that allow removing the antibiotic resistance gene using Flp recombinase from plasmid pCP20 (42). After recombination, all λ-red insertions were confirmed by colony PCR and the alleles were moved into new strains by P1 phage transduction. Strains imaged with microfluidic-based microscopy constitutively express P_RNA1_-mKate2, inserted at the Tn7 locus to serve as a fluorescent cell marker. The dual reporter strain carrying the *P_ada_-CFP* reporter has been described in (37). It is a transcriptional fusion of the *ada* promoter followed by the fast-maturing CFP variant SCFP3A inserted at the chromosomal *intS* site (∼150 kb downstream from the native *ada* gene). The *ΔalkB* deletion strain was obtained from the Coli Genetics Stock Center (CGSC 9779) and moved into other strains by P1 phage transduction. The *ΔalkA* and *Δada-alkB* mutants were engineered by λ-red recombination using plasmids pKD3 (cat) and pKD4 (kan), respectively, as templates. The chromosomal *ada^C321A^* point mutant was engineered by λ-red recombination that replaced a *Δada-alkB* operon deletion using plasmid pMV010. This plasmid was synthesized with GeneArt Gene Synthesis (ThermoFisher Scientific) and site-directed mutagenesis. It carries the *P_ada_-ada^C321A^-alkB* operon where the codon encoding Ada C321 was replaced with A321. A chloramphenicol resistance cassette was inserted downstream of *alkB* into pMV010 to select for recombinant cells after λ-red recombination into *Δada-alkB*. Lists of strains and primers used in this study are shown in Supplementary Tables S1 and S2.

### Cell culture

Strains were streaked from frozen glycerol stocks on to LB agarose with appropriate antibiotic selection. A single colony was used to inoculate each LB culture and grown for 6–7 h at 37°C. The cultures were then diluted 1:1000 into supplemented M9 minimal medium containing M9 salts (15 g/l KH_2_PO_4_, 64 g/l Na_2_HPO_4_, 2.5 g/l NaCl, 5.0 g/l NH_4_Cl), 2 mM MgSO_4_, 0.1 mM CaCl_2_, 0.5 μg/ml thiamine, MEM amino acids, 0.1 mg/ml L-proline and 0.2% glucose. Cultures were grown overnight to stationary phase, then diluted 1:50 into supplemented M9 medium and grown to OD_600_ = 0.2 before performing the experiments described in the following. Cultures were treated with methyl methanesulfonate (MMS, Sigma) as indicated in the figure legends. For microfluidic-based experiments, cells were grown for approximately 5 h before MMS addition to allow them to adapt to growth conditions in the microchannels.

### Single-molecule counting microscopy

Cells expressing HaloTag fusions were labelled with TMR ligand (Promega) following the procedure previously described in (41). Briefly, 1 ml of cell culture was concentrated by centrifugation and resuspended into 100 μl of supplemented M9 minimal medium. 5 μl of 2.5 μM TMR ligand was added to the cell resuspension and incubated for 30 min at 25°C. Free dye was removed with four rounds of washing. Cells were resuspended into 1 ml of supplemented M9 minimal medium and incubated at 37°C for 30 min. Cells were pelleted and resuspended into 2.5% paraformaldehyde in PBS buffer and fixed for 30 min at room temperature. Fixed cells were centrifuged, concentrated 10-fold and 1 μl of the cell resuspension was spotted on an agarose pad. Single-molecule imaging was performed using a custom-built total internal reflection fluorescence (TIRF) microscope under oblique illumination at room temperature. To reduce both background noise and the probability of missed detection due to TMR blinking, we used 1 s exposure time and acquired five frames per field of view under continuous 561 nm excitation at 0.2 kW/cm^2^. The five frames were then averaged and fluorescent spots were counted manually using cell outlines detected in transmitted illumination bright-field images.

### Single-cell microfluidic-based microscopy

The microfluidic single-cell imaging device was previously designed and experiments were performed as described in (39). About 0.85 mg/ml of surfactant pluronic F127 (Sigma Aldrich) was added to cultures to avoid cell aggregation in the microfluidic device. In addition to fluorescent reporters of the adaptive response, strains used for single-cell measurement constitutively expressed the fluorescent protein mKate2 and carried an *flhD* gene deletion to remove flagellum motility. Imaging was performed on a Nikon Ti Eclipse inverted fluorescence microscope equipped with perfect focus system, 100× NA1.45 oil immersion objective, sCMOS camera (Hamamatsu Orca Flash 4), motorized stage, an LED excitation source (Lumencor SpectraX) and 37°C temperature chamber (Okolabs). Fluorescence images were automatically collected using NIS-Elements software (Nikon) at 3-min intervals with exposures time of 75 ms for msCFP3, 100 ms for mKate2 and 300 ms for mYPet, using 50% LED excitation intensities.

Microscopy movies were analyzed using custom MATLAB software to segment cells based on cytoplasmic mKate2 fluorescence. Only mother cells at the end of each channel were included in the analysis. Cell deaths were manually detected when growth ceased, or when time traces terminated abruptly because cell filamentation led to the disappearance of the cell from the growth channel. The proportion of cells that exhibit lysis, growth arrest or escape microchannels due to filamentation is reported in the supplementary data (Supplementary Figure S7). mYPet and CFP reporters intensities were calculated from the average pixel intensities inside the segmented cell area and subtracting the background signal outside of cells. Detection of MutL-mYPet foci for DNA mismatch rate determination was performed with a spot-finding algorithm (43). When foci persisted for several frames, only the first frame was counted as a DNA mismatch event. DNA mismatch rates were calculated by dividing the number of observed MutL-mYPet foci events in each frame by the observation time interval (3 min) and the number of observed cells. Pearson correlation coefficients were calculated using the MATLAB corrcoef function. Cross-correlations between fluorescence signals were calculated using the MATLAB xcorr function. Normalized fluorescence traces of CFP and mYPet were obtained from the fluorescence intensity traces averaged over all observed cells and divided by its maximum value.

### Fluorescence-activated cell sorting (FACS) and mutation frequency experiments

4 ml of M9 supplemented with kanamycin (25 μg/ml) was inoculated with a single colony of WT (SU828) or *Δada-alkB* (SU829). Here, cells were not pre-cultured overnight to limit the MMS-independent emergence of mutation conferring rifampicin resistance. SU828 and SU829 contain a constitutive mKate2 segmentation marker and a kanamycin resistant plasmid (pUA139) encoding a *P_ada_*-*gfpmut2* reporter (obtained from (44)). At OD_600_ = 0.2, cells were treated with 1, 3 or 10 mM MMS for 90 min. 1 ml of cells were then washed two times by centrifugation and resuspended into 5 ml PBS to remove residual MMS. Cells were sorted and analysed with a S3e™ Cell Sorter and ProSort™ Software (BioRad). Fluorescence intensities of cells were measured using 488 and 561 nm lasers. Signals were collected using the emission filters FL1 (525/30 nm) and FL3 (615/25 nm) for GFPmut2 and mKate2, respectively. Voltages of the photomultipliers were 500, 300, 600 and 720 volts for FSC (forward scatter), SSC (side scatter), FL1 and FL3, respectively. Histograms were gated to sort the activated and delayed cell populations. The Del sorting gate was defined on a culture that had not been treated with MMS. The activated sorting gate had the same size as the Delayed sorting gate but centred at the maximum of the GFP peak (Supplementary Figure S8). Cells outside or in between the two gates were excluded from the sorting procedure. Cells were sorted in 5 ml PBS at a rate of 100 000 s^–1^. Sorted cells were diluted into 10 ml of LB and incubated at 37°C for 1 h, concentrated by centrifugation and resuspended into 1 ml LB before plating on freshly prepared LB agar plates with 20 μg/ml rifampicin. After overnight incubation at 37°C, the number of rifampicin resistant colonies was counted and divided by the number of sorted cells to obtain the mutant frequency. Measurements were carried out three times from independent starting cultures.

## RESULTS

### Stochastic activation of *ada* propagates across the whole adaptive response regulon

Alkylation stress causes mutagenic DNA lesions that promote error-prone DNA replication, and toxic lesions that block DNA replication forks and lead to cell death if left unrepaired (35). Indeed, *E. coli* strains with deletions of individual genes of the adaptive response, namely *ΔalkA, ΔalkB* and *Δada-alkB* (lacking the entire *ada-alkB* operon), were unable to grow on plates in the presence of the alkylating agent methyl methanesulfonate (3 mM MMS) that causes both mutagenic and toxic lesions (Supplementary Figure S1) (35). However, deletion of *aidB* did not affect cell survival (Supplementary Figure S1). Considering the importance of the adaptive response for tolerance of alkylation stress, it is surprising that the master regulator Ada is so sensitive to gene expression noise that its feedback autoregulation generates large variation in Ada abundances across cells in a population after MMS treatment (37). Notably, stochastic activation of the adaptive response is conserved across different *E. coli* strain backgrounds (AB1157, MG1655 and MC4100) (Supplementary Figure S2). As AlkA and AlkB are crucial for survival of alkylation damage (Supplementary Figure S1), we asked whether the stochastic activation of *ada* also impacts their expression. In principle, variation in the master regulator could be buffered or propagated in the gene regulatory network. To this end, we monitored the endogenous expression of functional *ada, alkB, alkA* and *aidB* translational fusions to the fluorescent protein mYPet (Supplementary Figure S1) at the single-cell level in a microfluidic device. The ‘mother machine’ setup allows imaging hundreds of single cells over tens of generations under constant growth conditions and defined stress treatments (10,39,45). We observed that the addition of MMS in the fluidic system caused most cells to activate the adaptive response regulon rapidly (termed ‘Activated’ state) (Figure 1C–E). However, we detected a fraction of cells that delayed the activation of AlkB and AlkA expression for the duration of multiple cell cycles (termed ‘Delayed’ state), despite constant treatment with a fixed concentration of MMS (Figure 1D, E and Supplementary Figure S3). This cell-to-cell heterogeneity in *alkB* and *alkA* gene induction matches the patterns seen for *ada* (Figure 1C–E) (37). We did not detect any activation of *aidB* expression in response to MMS (Figure 1F). It is thus likely that *aidB* is not expressed in the conditions of our experiments. Indeed, the role of *aidB* in DNA repair has been brought into question before (35,46,47), and its contribution to the alkylation stress response appears to be negligible considering that a *ΔaidB* strain has the same MMS sensitivity as the wild-type (Supplementary Figure S1).

### Fluctuations in *ada* expression are accurately propagated to *alkA*

**Figure 2.**
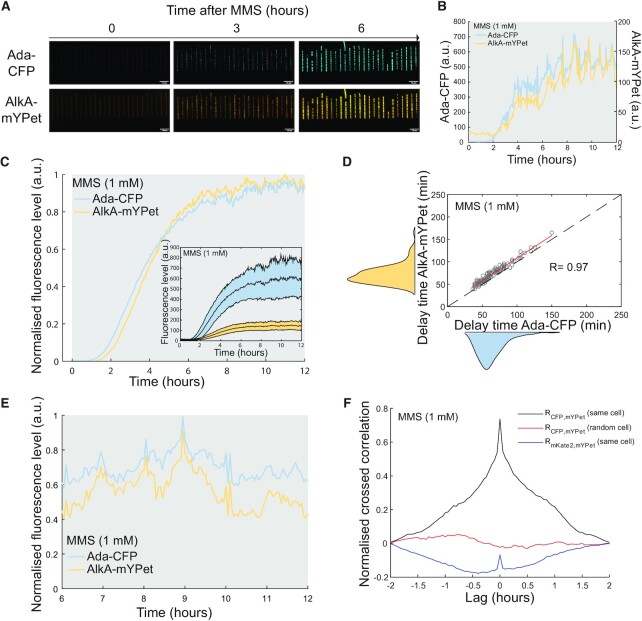
Fluctuations in *ada* expression are propagated to *alkA*. Dual reporter assays of Ada-CFP and Alka-mYPet expression. (**A**) Example snapshots of microfluidic single-cell imaging of the dual reporter strain carrying Ada-CFP (cyan) and AlkA-mYPet (yellow) reporters with constant 1 mM MMS treatment. (**B**) Example time traces showing activation of Ada-CFP and AlkA-mYPet after 1 mM MMS addition (shaded background) in a single cell. (**C**) Cell-average fluorescence intensity of Ada-CFP and AlkA-mYPet (cells = 139). Curves were normalized by their maximum value and background level at time of MMS addition (0 h) was subtracted. Inset shows fluorescence time traces and their standard deviations about the mean without normalization. (**D**) Correlation plot showing the delay between 1 mM MMS addition and response activation for Ada-CFP and AlkA-mYPet. Each circle represents one cell (cells = 139). R: Pearson correlation coefficient. Average delays Ada-CFP = 63 ± 19 min (standard deviation), AlkA-mYPet = 71 ± 20 min (standard deviation). The red line shows the best linear fit (AlkA delay = Ada delay + 8 min). (**E**) Example single-cell trace showing correlated fluctuations of Ada-CFP and AlkA-mYPet expression at steady-state after response activation with constant 1 mM MMS treatment. (**F**) Cross-correlation analysis of Ada and AlkA expression at steady-state after response activation with constant 1 mM MMS treatment. Cross-correlation curves were computed from the Ada-CFP and AlkA-mYPet intensity traces of individual cells, and then averaged over 139 cells (black curve). The expression dynamics of the two genes are positively correlated over a relative lag period between the signals of ± 1 h. As controls, there is no correlation between AlkA-mYPet and the intensity of the constitutively expressed mKate2 cell marker (blue curve), and no correlation between Ada-CFP and the AlkA-mYPet intensity from a different randomly chosen cell (red curve). Controls were also averaged over 139 cells. Therefore, correlations between Ada and AlkA expression are not caused by global fluctuations in cell growth behaviour or the measurement conditions.

The similarity of *ada* and *alkB* activation timing was expected because both genes are in the same operon; however, the variability observed for *alkA* activation was less anticipated since both unmethylated and methylated forms of Ada have been proposed to activate *alkA* (48,49). Thus, to precisely quantify the activation times of *ada* and *alkA* in the same cell, we engineered dual reporter strains expressing endogenous Ada-CFP and AlkA-mYPet fusions (Figure 2A,B and Supplementary Figure S1). We observed that both *ada* and *alkA* expression share highly correlated activation times (Figure 2C and D). To confirm that the translational CFP fusion to Ada did not affect our observation, we monitored AlkA-mYPet and an ectopic transcriptional P_ada_-CFP fluorescent reporter. In this strain, the endogenous *ada* allele is unaltered and the activation of AlkA and P_ada_ is almost simultaneous (Supplementary Figure S4). We further noted that *ada* and *alkA* both displayed broad fluctuations in expression level in single cells with constant MMS treatment even when the cell-average expression had reached steady-state after the period of response activation (Figures 2B, C and E). We previously showed that the steady-state fluctuations of Ada reflect variation in the amount of DNA damage in individual cells over time (37). Temporal cross-correlation between Ada-CFP and AlkA-mYPet signals showed that fluctuations of *ada* expression are correlated with those of *alkA* at a single-cell level (Figure 2F). As a control, we did not detect cross-correlations between Ada-CFP and AlkA-mYPet signals from different random cells or between AlkA-mYPet and the P_RNA1_-mKate2 constitutive fluorescent reporter (Figure 2F).

It has been shown that a C321A substitution in Ada increases its expression in the absence of alkylation stress (36,50,51). At the single-cell level, we indeed found that cells carrying the *ada^C321A^* variant spontaneously activate *ada* expression before MMS treatment (Supplementary Figure S5). Yet, the *ada^C321A^* variant did not affect *alkA* expression in the absence of alkylation stress (Supplementary Figure S5). Upon MMS treatment, however, *alkA* expression was no longer stochastic but was activated rapidly and homogeneously in all *ada^C321A^* cells (Supplementary Figure S5). Hence, while Ada C321A substitution is sufficient to trigger *ada* expression, the methylation of Ada-N remains necessary for induction of *alkA*. Consistent with the low expression of *ada* being the source of noise in the response, the elevated basal level of Ada^C321A^ abolishes the heterogeneity in *alkA* induction.

### The basal level of the adaptive response proteins is low and heterogeneous

**Figure 3.**
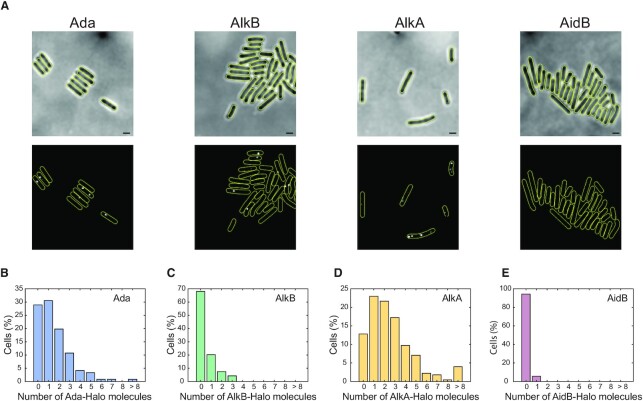
The basal expression level of the adaptive response proteins is very low. (**A**) Example of single molecule spots detected within chemically fixed cells after *in vivo* HaloTag labelling with TMR ligand (without MMS treatment). Upper panel = brightfield, lower panel = TMR fluorescence; scale bar = 1 μm. The distribution of Ada-Halo (cells = 121), AlkB-Halo (cells = 94), AlkA-Halo (cells = 238) and AidB-Halo (cells = 105) proteins per cell are shown in panels (**B–E**), respectively.

The propagation of stochastic *ada* activation to the whole response regulon means that the cells with a delayed response (the Delayed subpopulation) dwell in a state in which all proteins of the Ada regulon are only expressed at a basal level. We therefore quantified the basal expression of these proteins using a method to count translational protein fusions to the HaloTag, which can be labelled with the fluorescent ligand TMR (26). MMS sensitivity assays confirmed the functionality of the *ada, alkA* and *alkB* HaloTag fusions (Supplementary Figure S1). Chemical fixation of cells allowed us to capture long camera exposures on a custom-built single-molecule fluorescence microscope in order to detect distinct fluorescent spots and count protein copy numbers per cell (Figure 3A). The basal expression of Ada was previously shown to be as low as 1 molecule/cell on average (37,52), which is similar to the distribution of Ada-Halo molecules/cell that we observed here (Figure 3B). Single-molecule counting of AlkB-Halo revealed that most cells were completely devoid of AlkB in absence of alkylation stress (Figure 3C). Only ∼20% of the population exhibited a single AlkB protein (Figure 3C). This observation is surprising given the importance of AlkB for the repair of alkylation damage (Supplementary Figure S1); however, it is not unexpected considering that *alkB* is positioned at the end of the *ada-alkB* operon. We further quantified the absolute number of AlkA-Halo proteins (Figure 3D). Although some cells (∼5% of the population) contained too many proteins (>8) to be accurately counted, most cells in the population exhibited a low number of AlkA, with ∼2.6 molecules per cell on average. As for AlkB, it is surprising that the important DNA repair protein AlkA is expressed at such low levels. Of note, we did not detect any AidB-Halo proteins in most cells (>95% of the population) (Figure 3E). The distributions of Ada, AlkB and AlkA copy numbers were well described by Poisson distributions with Fano factors (variance/mean) close to 1, indicating that these proteins are produced at a constant rate without bursting (Supplementary Figure S6). Overall, our results demonstrate that AlkA and AlkB are necessary for the cell to survive alkylation stress (Supplementary Figure S1), yet they are present at very low level and in many cells completely absent before induction.

### Contribution of Ada, AlkB and AlkA to genome maintenance

**Figure 4.**
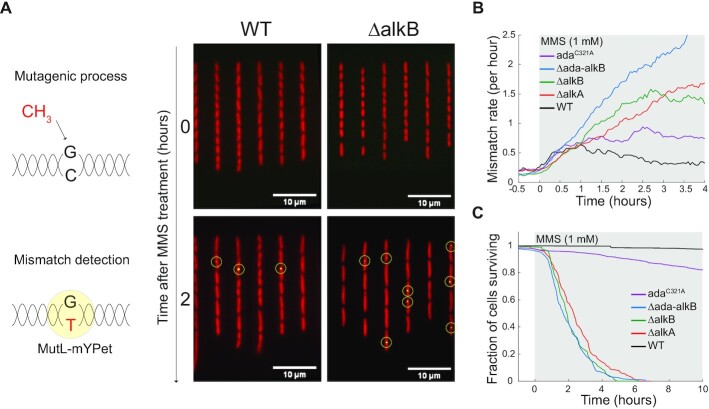
Contribution of Ada, AlkB and AlkA to cell survival and mutation prevention during alkylation stress. (**A**) Example of real-time imaging of DNA mismatches. DNA methylation lesions result in nucleotide misincorporation during DNA replication. DNA mismatches are recognized by MutL-mYPet that forms fluorescent foci (yellow dots) and enables automated DNA mismatch detection (yellow circles). Fluorescence of the segmentation marker mKate2 is shown in red. (**B**) Cell-average rate of DNA mismatch foci during constant 1 mM MMS treatment (shaded background) for strains *Δada-alkB* (blue, cells = 435), *ΔalkB* (green, cells = 347), *ΔalkA* (red, cells = 518), *ada^C321A^* (purple, cells = 395) and the WT strain (black, cells = 527). Mismatch rate curves have been smoothed using a moving average filter of 30 min. (**C**) Distribution of cell survival times in the microfluidic channels during constant 1 mM MMS treatment for the same strains.

Whether heterogeneity in the adaptive response has a beneficial function for a cell population remains an open question. An interesting hypothesis is that the delay of Ada, AlkB and AlkA activation could increase the mutation rate of certain cells and therefore increase genetic plasticity of the population (53). We previously showed that cells with a delayed adaptive response have a higher rate of DNA replication errors during MMS treatment than cells that rapidly activated the response (37,39). To address the contribution of each component of the adaptive response regulon to genome maintenance under alkylation stress, we applied a method that enables the detection of nascent DNA replication errors. We used strains expressing fluorescently labelled MutL-mYPet fusion proteins that form distinct foci when bound at DNA mismatches (Figure 4A) (10,39). As shown before, delayed activation of the adaptive response causes a transient burst in the rate of DNA mismatches that lasts for ∼2 h after the addition of 1 mM MMS (39). Unlike the wild-type, strains with the gene deletions *ΔalkB*, *ΔalkA* and *Δada-alkB* all showed elevated and sustained mismatch rates that did not recover during prolonged MMS treatment (Figure 4B). Addressing the specific function of Ada in DNA repair is more complex than for AlkB and AlkA because Ada has a dual role as an O^6^meG repair protein and regulator of the adaptive response. To separate these functions, we relied on the endogenous chromosomal Ada mutant, Ada^C321A^, that lacks the catalytic cysteine required for repair of O^6^meG lesions (Supplementary Figure S1). This mutant is still able to activate the adaptive response (Supplementary Figure S5) (36,50,51). Upon alkylation stress, O^6^meG repair deficiency resulted in a sustained and increased mismatch rate with respect to the WT level but remained below the levels of the *ΔalkB* and *ΔalkA* strains (Figure 4B). Therefore, AlkB, AlkA and Ada each provide specific DNA repair functions that are important for mutation prevention during alkylation stress.

### Contributions of Ada, AlkB and AlkA to cell survival

Although mutagenesis is essential for genome evolution, individual mutant cells that emerge during stress still need to survive in order to propagate their genetic innovations. We thus examined cell survival during MMS treatment by monitoring cell elongation and division in the microfluidic chips. Cell death manifested as sudden lysis or complete cessation of growth (Supplementary Figure S7). A fraction of cells grew into long filaments that escaped from the growth channels (Supplementary Figure S7). Despite the delay in the induction of alkylation repair, cell survival of the wild-type strain was essentially unaffected at 1 mM MMS (Figure 4C), owing to the constitutively expressed DNA glycosylase Tag and DNA damage tolerance pathways that are controlled by the SOS response (39). This was not the case for the *ΔalkB*, *ΔalkA* and *Δada-alkB* deletion mutants, with <10% of cells surviving after 4 h of constant MMS treatment for each of these strains (Figure 4C and Supplementary Figure S7). This result indicates that beyond a certain level of 3meA lesions (repaired by AlkA), and 3meC and 1meA lesions (repaired by AlkB), alternative repair and damage tolerance pathways cannot compensate for the lack of AlkA and AlkB. Prolonged failure to repair these lesions leads to DNA replication stalling (35,54,55), a process that is ultimately lethal to cells. On the other hand, 90% of *ada^C321A^* cells were alive after 4 h of constant MMS treatment, showing that Ada’s repair function protects predominantly against the mutagenic effects of alkylation stress rather than its toxicity.

### Cell-to-cell heterogeneity in the adaptive response leads to differences in genomic mutation rates

**Figure 5.**
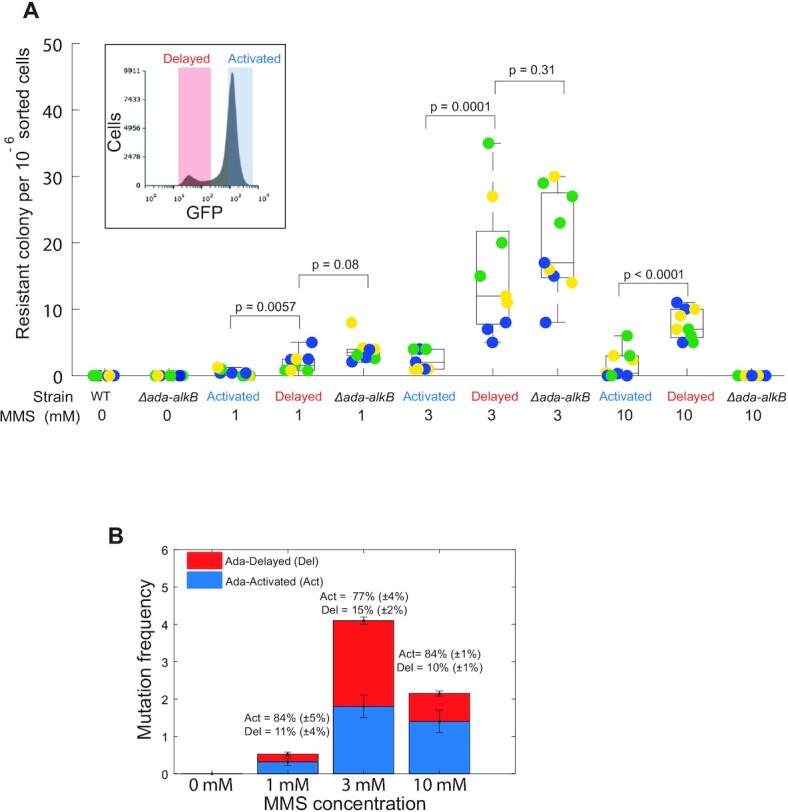
Increased mutation frequency of the cell subpopulation with a delayed adaptive response. (**A**) Boxplots showing the number of rifampicin-resistant colonies for Ada-Activated, Ada-Delayed subpopulations and the *Δada-alkB* strain after 90 min treatment with different MMS concentrations. Each subpopulation was sorted according to sorting gates defined by P_ada_-GFP intensity (example in inset). Biologically independent experiments (cultures started from distinct singles colonies) are grouped by colour. For each biological replicate, three rounds of sorting were performed and plated on separate rifampicin plates. *P*-values from two-tailed *t*-test. (**B**) Barplot showing the frequency of rifampicin-resistance mutations of Ada-Activated and Ada-Delayed subpopulation after 90 min treatment with different MMS concentrations. The mutation frequency was computed from the product of the rifampicin-resistant mutant counts and the percentage of cells sorted for each subpopulation (average percentages shown).

Our mismatch rate measurements imply that phenotypic heterogeneity in the adaptive response causes cell-to-cell variation in mutation rates. Indeed, most DNA mismatches are repaired by the MMR system, but ∼1% are overlooked and turn into stable mutations in the next round of replication (56). However, whether differences in DNA mismatch rates truly reflect a genuine variation in mutation rates between cells remains unknown. To address this important point, we used fluorescence-activated cell sorting (FACS) to distinguish and collect cells that differentially activated the adaptive response after MMS exposure. We used a plasmid-based *P_ada_*-*gfpmut2* reporter for the adaptive response that allowed us to distinguish between the Activated and Delayed subpopulations of cells after MMS treatment (Supplementary Figure S8). We sorted an identical number of 10^6^ cells from the two subpopulations and measured their respective mutation frequencies based on the number of colonies resistant to the antibiotic rifampicin (Figure 5A). We found that the mutation frequency was significantly higher for the Delayed than the Activated subpopulations after 90 min of treatment with 1 mM (∼1.5-fold difference), 3 mM (∼5-fold difference) and 10 mM MMS (∼4-fold difference) (Figure 5A). Therefore, cell-to-cell variation in the timing of the adaptive response indeed causes substantial differences in genomic mutation rates. These results also confirm that the detection of MutL-mYPet foci as markers for DNA mismatches reports on the genomic mutation rates of single cells (10,39).

### Elevated mutation frequency of the Delayed subpopulation compared to *Δada-alkB* cells at high stress levels

To confirm that the *P_ada_-gfpmut2* reporter activation was dependent on Ada, we also performed FACS with *Δada-alkB* cells. As expected, *P_ada_-gfpmut2* remained inactivated independently of the MMS concentration (Supplementary Figure S8). Furthermore, the mutation frequency of *Δada-alkB* cells was similar to that of the Delayed subpopulation at 1 or 3 mM MMS. Therefore, wild-type cells that fail to activate the Ada response because of gene expression noise suffer the same mutagenic effects of alkylation stress as cells that lack the *ada-alkB* operon completely. However, *Δada-alkB* cells differed strongly from the Delayed subpopulation at the higher dose of 10 mM MMS (Figure 5A). We did not detect any rifampicin-resistant colonies for the *Δada-alkB* strain after 10 mM MMS treatment, whereas the Delayed subpopulation generated a significant number of such colonies (Figure 5A). We attribute the lack of mutant colonies to the extremely low survival of *Δada-alkB* cells in the presence of MMS. Thus, although *Δada-alkB* deletion promotes alkylation-induced mutagenesis (Figure 4B), it also rapidly increases the likelihood of cell death (Figure 4C). The disproportionate effect of the *Δada-alkB* deletion on the population dynamics diminishes overall evolvability at high stress levels. Although Delayed cells initially behave like *Δada-alkB* cells, they are capable of activating the Ada response eventually. This enables the repair of toxic DNA lesions that are otherwise lethal in *Δada-alkB* cells. The Delayed subpopulation therefore accumulates mutations during the adaption lag but maintains chances of survival after response activation. These features make the Delayed subpopulation a pool of increased genetic plasticity.

We finally sought to address whether the Delayed subpopulation contributes significantly to the genetic plasticity of the whole population. This depends on several characteristics of the subpopulation, namely its size, mutation rate and viability. First, we measured the generation times of the Delayed and Activated cells and obtained similar results (Supplementary Figure S9), ruling out the possibility that the difference in mutation frequency could be caused by an unequal number of cell divisions of each subpopulation. Then, by quantifying the number of rifampicin-resistant colonies relative to the abundances of the subpopulations, we found that the Delayed subpopulation generates a substantial fraction of all viable mutants despite its small size (Figure 5B). This analysis also demonstrated that genetic plasticity is a product of mutability and survival. The Activated and Delayed subpopulations both have a basal DNA damage tolerance owing to constitutively expressed DNA repair pathways and the SOS response. Increasing stress leads to higher mutation frequency, but the lack of inducible DNA repair capacity in the Delayed subpopulation means that cell survival drops disproportionately as the damage level rises. Because of this, the effect of the Delayed subpopulation is maximal at intermediate damage level (3 mM MMS), where the subpopulation of 15% of cells generates 53% of the total rifampicin-resistant mutants.

## DISCUSSION

Because emergence of mutations in a cell population is driven by rare and stochastic molecular events, mechanisms governing this process can be lost in the averaged result commonly gained from bulk experiments. We thus used a single-cell approach to study how regulatory dynamics of DNA repair genes influence mutation and cell survival, and ultimately impact genetic plasticity of a cell population. Focusing on the adaptive response to DNA alkylation stress in *E. coli*, we found that, with the exception of *aidB*, the whole adaptive response regulon (i.e. *ada, alkB* and *alkA*) is heterogeneously activated across isogenic cells during alkylation stress.

Despite the apparent noisiness of the adaptive response, our results demonstrate that it is in fact a remarkably precise gene regulatory network that splits an isogenic population of cells into two defined subpopulations that coexist for several hours with distinct gene expression states. The production of a single Ada molecule combined with positive feedback amplification functions as the stochastic master switch in this network. The random timing of Ada activation in each cell is precisely transmitted to induce AlkB and AlkA expression (Figures 1 and 2). Interestingly, Ada levels are upregulated 1000-fold in response to alkylation damage, yet cells expressing the non-functional *ada^C321A^* mutant that is defective in O^6^meG lesion repair are not sensitized to alkylation damage and exhibit only slightly increased mismatch rates. The benefit of very high Ada numbers after response activation could be an increased robustness to gene expression noise. Indeed, we found that fluctuations in *ada* expression are accurately propagated to *alkA*.

Cells with delayed Ada activation are essentially devoid of all adaptive response proteins because of their extremely low basal expression levels (Figure 3). Therefore, the Delayed state is distinct and defined not just by the absence of the Ada regulator, but an all-round lack of proteins that are crucial for DNA alkylation repair. After switching to the Activated state, fluctuations in Ada production are propagated such that the whole response regulon (except *aidB*) closely follows the state of the regulator (Figures 1 and 2). These findings suggest that the stochastic phenotypic heterogeneity generated by the adaptive response is an evolved property of the system rather than a side-effect of a regulatory inaccuracy. This view is further supported by several lines of evidence.

First, at the core of the Ada response is a positive feedback loop, where methylation of Ada induces its own expression. In general, positive feedback amplifies variation, and the Ada system represents an extreme case of this where cells with zero copies of Ada cannot trigger the feedback loop at all (38). Second, deactivation of the response after removal of alkylation stress is completely uniform across cells (37,38). This shows that cells are capable of sensing the stress level and regulating the response accurately. Third, the heterogeneity and delays in the response can be avoided by genetic modifications that cause either a slight increase (37) or constitutive Ada expression (Supplementary Figure S5). The fact that these changes are not present, suggests that the heterogeneity and activation delays have a functional effect. Fourth, lack of AlkB and AlkA expression is very toxic to cells in the presence of alkylation stress (Figure 4 and Supplementary Figure S1), suggesting that the formation of a subpopulation of cells in which these proteins are absent has a purpose that outweighs the fitness costs. Fifth, we had previously considered the possibility that Ada and AlkA levels are very low because the proteins are toxic at high expression levels (37,55). The response heterogeneity would then be a direct consequence of inevitable noise associated with the pressure to keep molecule numbers low. However, AlkB is not known to have any toxic effects, yet it is also present at very low numbers (Figure 3). Furthermore, the *ada^C321A^* mutant that leads to constitutive expression and rapid activation of AlkA does not show obvious fitness defects. Sixth, heterogeneous Ada induction is conserved across different *E. coli* strain backgrounds (Supplementary Figure S2). The RpoS sigma factor contributes to *ada* regulation (57), but the response heterogeneity was robust to the absence of RpoS in *E. coli* AB1157 (58) as compared to MG1655 and MC4100 strains that express functional RpoS. Notably, the close homologue of Ada in eukaryotes, MGMT, has been shown to be expressed heterogeneously across cells in glioblastoma tumours. This variation causes cell-to-cell differences in mutation rates that have been linked to the evolution of chemotherapy resistance (59–61).

**Figure 6.**
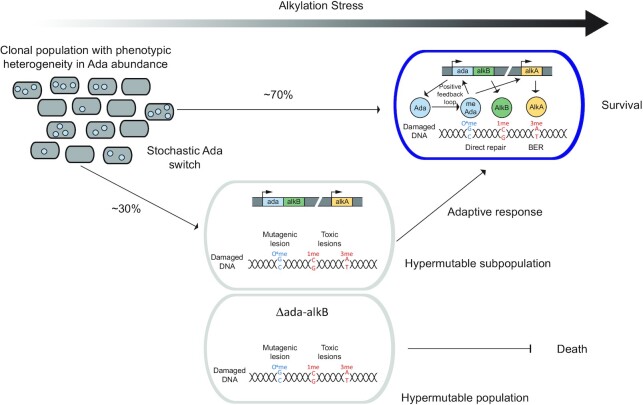
Cell-to-cell variability in DNA alkylation repair as a source of genetic plasticity. The stochastic expression of Ada splits the isogenic *E. coli* population into two distinct subpopulations. The subpopulation with a delayed adaptive response becomes hypermutable during alkylation stress. In contrast to a *Δada-alkB* hypermutable population, the delayed wild-type cells can eventually activate the adaptive response and thereby increase their chance of survival. This viable subpopulation can act as a source of genetic plasticity.

We speculate that the heterogeneity in the *E. coli* adaptive response represents the phenomenon of stress-induced mutagenesis, whereby cells poorly adapted to their environment increase their mutation rates (Figure 6). Whether this is an evolvability strategy *per se* or an unavoidable consequence of the selection for survival has been brought into question (17,62). Nonetheless, the functional benefit of stress-induced mutagenesis relies on the ability of cells to propagate any mutations that are generated during stress. Indeed, alternative DNA repair and damage tolerance mechanisms, such as constitutively expressed DNA glycosylases and the translesion synthesis DNA polymerases of the SOS response can rescue early failures to repair toxic alkylation lesions (39). However, when replication-stalling lesions saturate alternative repair strategies, the adaptive response becomes necessary for survival (39). Our study demonstrates that cells with a delayed adaptive response have an elevated mutation rate and maintain the capacity to propagate these mutations if they eventually activate the adaptive response. In this way, the transient hypermutable subpopulation generated by stochastic regulation of DNA alkylation repair could increase the genetic plasticity of the whole population.

## DATA AVAILABILITY

The data reported in this paper have been deposited in the Oxford University Research Archive: https://doi.org/10.5287/bodleian:7JmVb7zVb.

## Supplementary Material

gkab1143_Supplemental_File
