# Supplementary material for: Cellular heterogeneity in DNA alkylation repair increases population genetic plasticity
Source: Nucleic Acids Res. 2021 Nov 25;49(21):12320–31. doi: 10.1093/nar/gkab1143 (PMC8643705; doi:10.1093/nar/gkab1143)
Supplement: gkab1143_Supplemental_File [file gkab1143_supplemental_file.pdf]

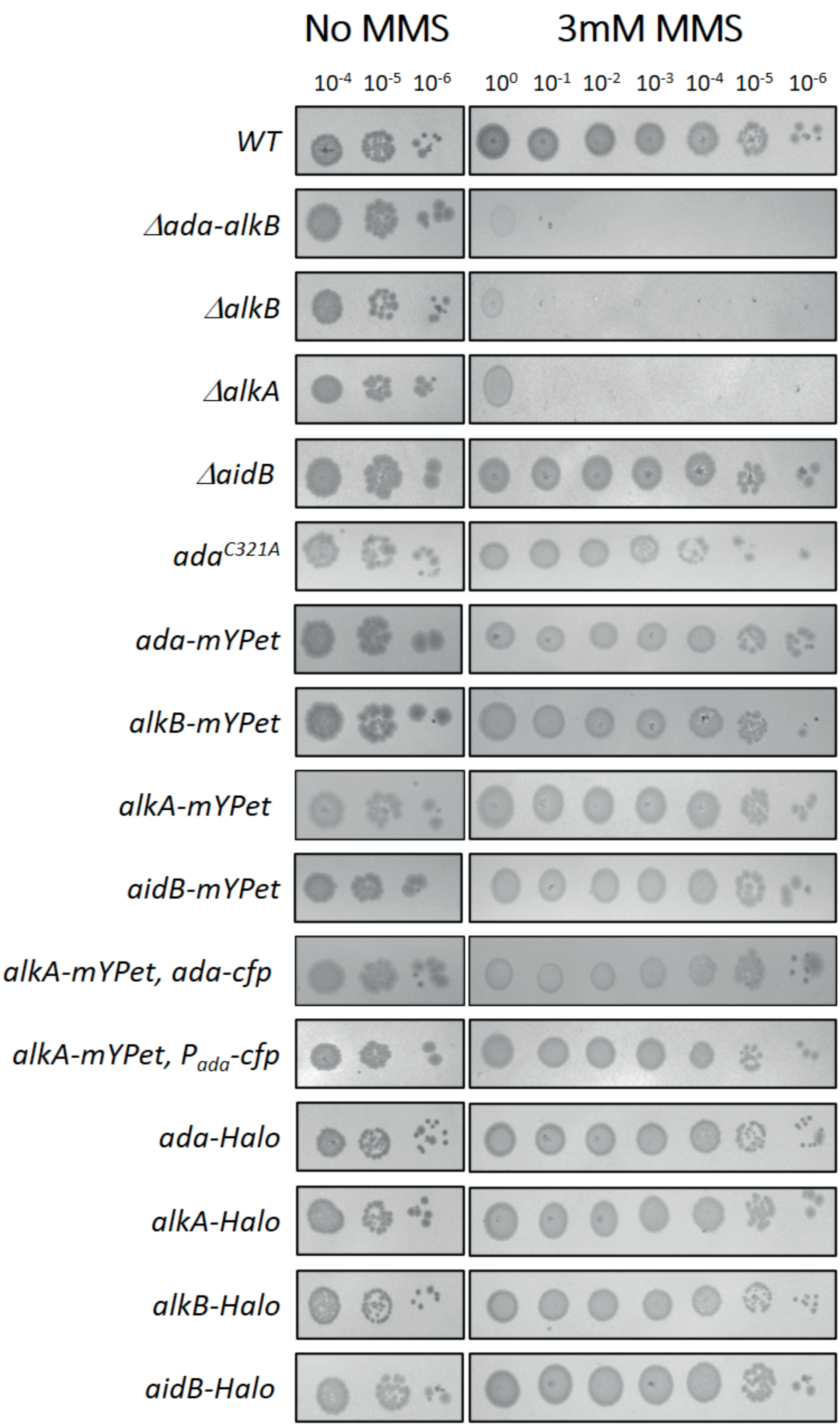

Supplementary Figure 1: MMS sensitivity assays

To assess the functionality of translational and transcriptional reporters used in this study, we performed MMS sensitivity tests by spotting 10-fold serial dilutions of over-night cell cultures on LB and LB + 3 mM MMS plates and compared growths after over-night incubation at 37C. Note that the P_{ada}-*cfp* reporter refers to an ectopic version of the *ada* promoter, the native P_{ada}-*ada-alkB* locus is unaltered in this strain.

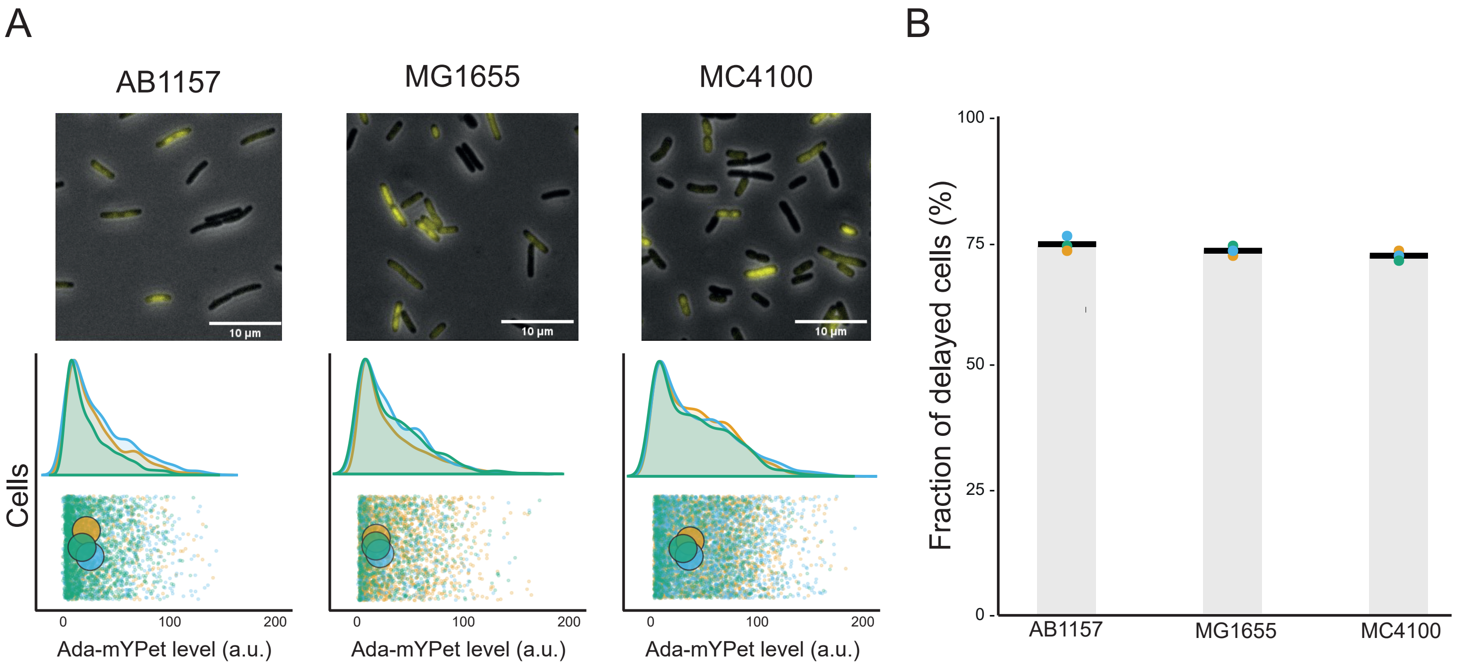

Supplementary Figure 2: Stochastic activation of the adaptive response is conserved in strains AB11157, MG1655 and MC4100

(A) Example snapshots showing heterogeneity in the adaptive response activation after 1 h of treatment with 1 mM MMS in strains AB1157, MG1655 and MC4100. Fluorescence level of cells (fluorescence background subtracted) carrying the Ada-mYPet reporter are plotted and grouped by colour to indicate independent biological replicates. Single-cell fluorescence intensities are represented below by small dots. Median intensities are indicated by large dots. For each strain, the distributions are skewed to the right due to the presence of cells that have induced Ada-mYPet expression. (B) Barplot showing the fraction of cells with delayed Ada-mYPet induction.

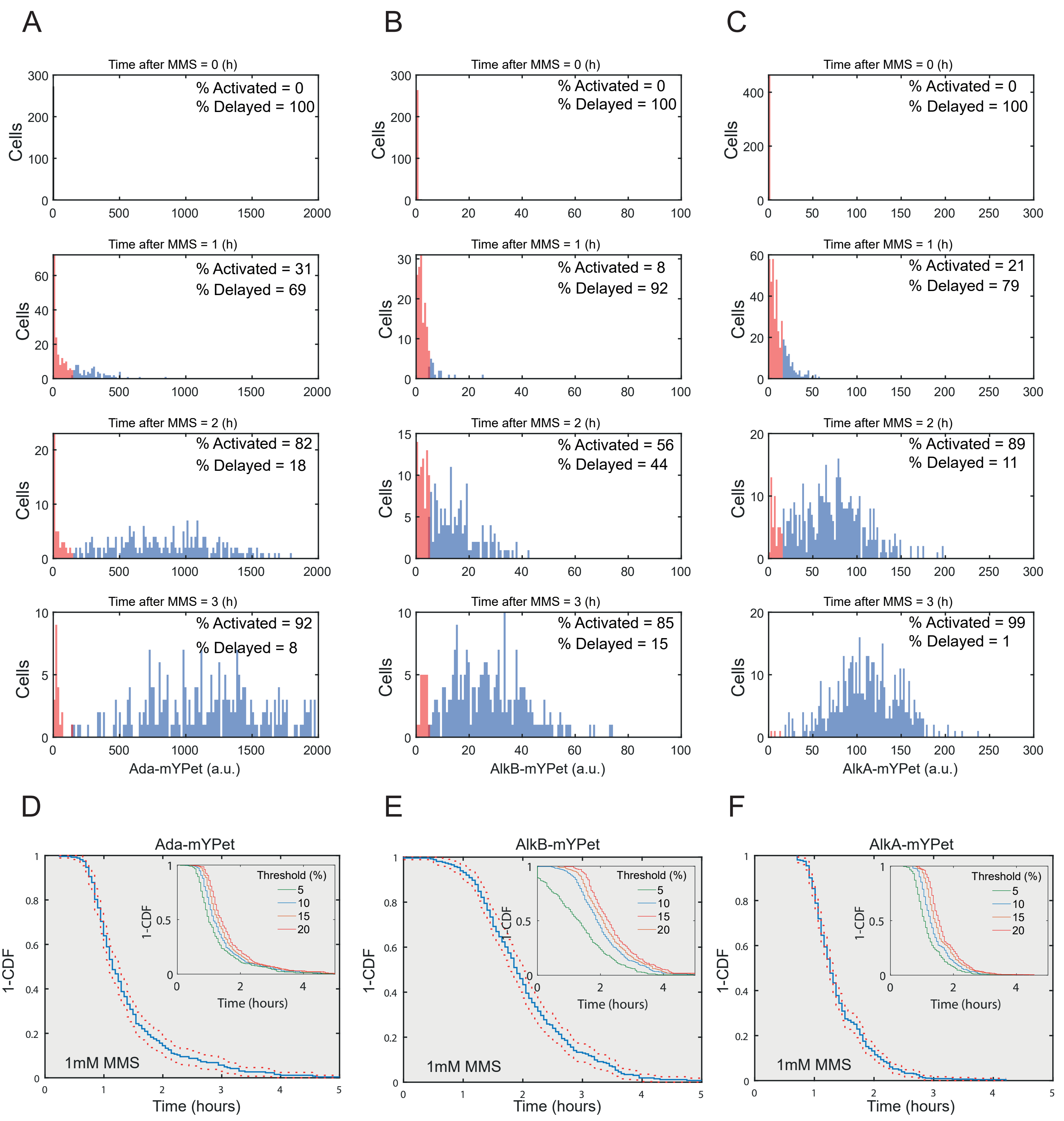

Supplementary Figure 3: Distributions of activated and delayed cells

Fluorescence intensity of cells carrying *ada-mYPet, alkB-mYPet* and *alkA-mYPet* treated with 1 mM MMS. The background intensity at time = 0 h was subtracted per cell. To quantify Activated and Delayed subpopulations, activation thresholds were defined as 10% of the maximum value of the cell-average intensity after activation (Ada = 145 a.u., AlkB = 5 a.u., AlkA = 16 a.u.). Histograms show Ada-mYPet (cells = 269) (A), AlkB-mYPet (294) (B) and AlkA-mYPet (cells = 459) (C) intensities (a.u.) after 0, 1, 2 and 3 hours after addition of 1 mM MMS treatment in the microfluidic device. Percentage of activated and delayed cells are shown. Activated cells are displayed in blue, delayed cells are displayed in red. (D-F) Cumulative distribution functions (1-CDF) show the decay in the fraction of cells with fluorescence intensity below the activation threshold for Ada-mYPet (D), AlkB-mYPet (E) and AlkA-mYPet (F) upon MMS treatment. Insets show 1-CDF for different activation thresholds (5, 10, 15 or 20% of the maximum value of the cell-average intensity after activation). Dashed red lines show 95% confidence bounds.

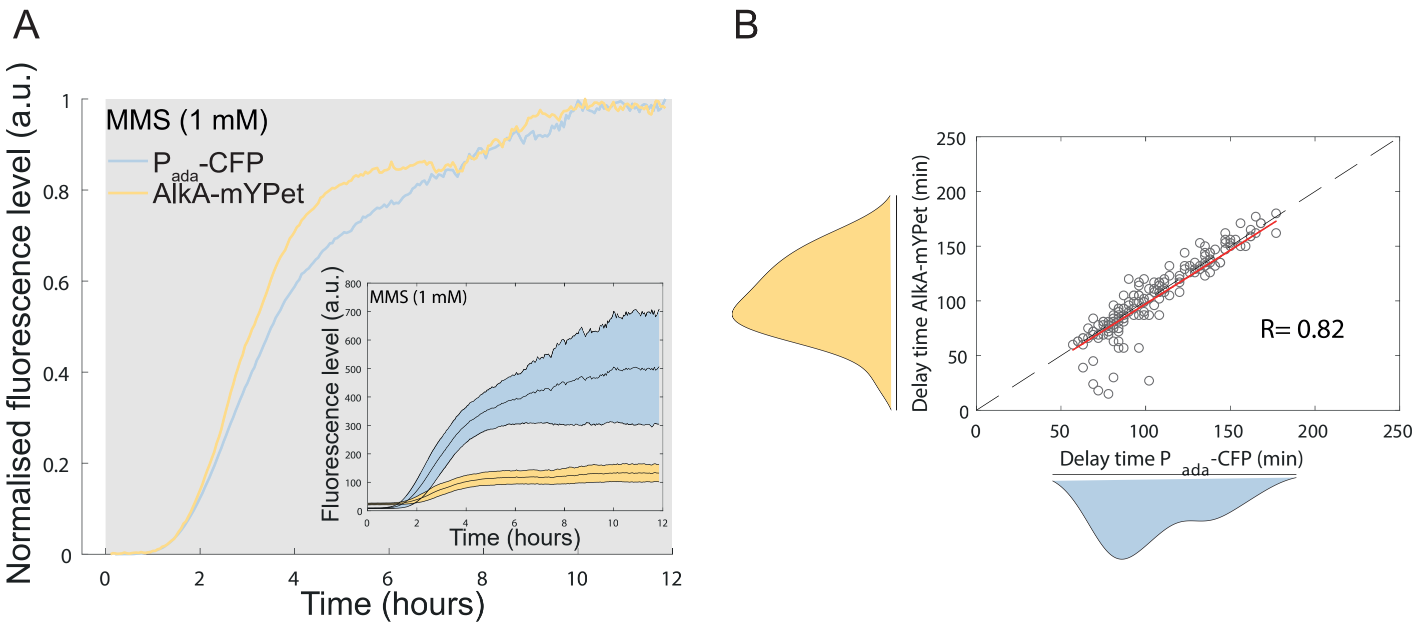

Supplementary Figure 4: Dual reporter assay of P_{ada}-CFP and AlkA-mYPet activation with 1 mM MMS treatment.

(A) Cell-average fluorescence intensity curves of P_{ada}-CFP and AlkA-mYPet (cells = 170). Curves were normalised by their maximum value and the background level at time of MMS addition (0 hours) was subtracted. Inset shows fluorescence time traces and their standard deviations about the mean without normalisation. (B) Correlation plot showing delay times between 1 mM MMS addition and response activation for P_{ada}-CFP and AlkA-mYPet. Each circle represents one cell. R: Pearson correlation coefficient. The red line shows the best linear fit.

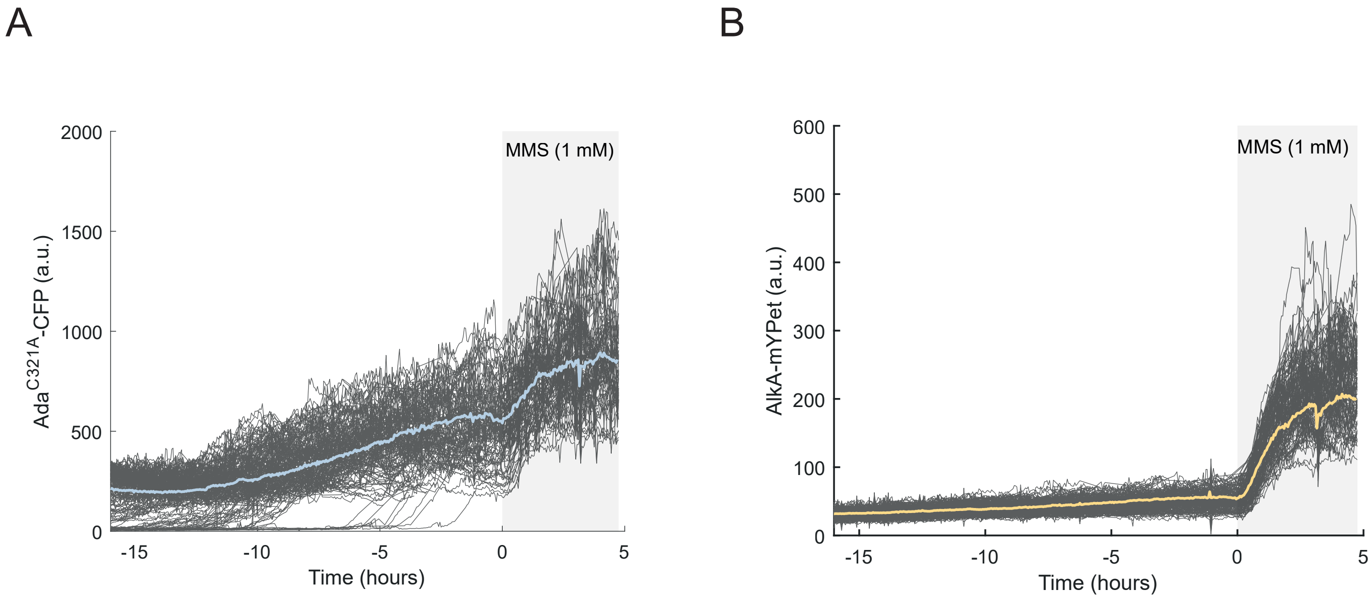

Supplementary Figure 5: Ada C321A substitution triggers ada auto-induction

Microfluidic-based imaging of the dual reporter strain carrying *ada*^{C321A}*-CFP* (A) and *alkA-mYPet* (B) (cells = 188). The shaded background indicates constant 1 mM MMS treatment. Coloured curves represent the cell average fluorescence intensity time trace.

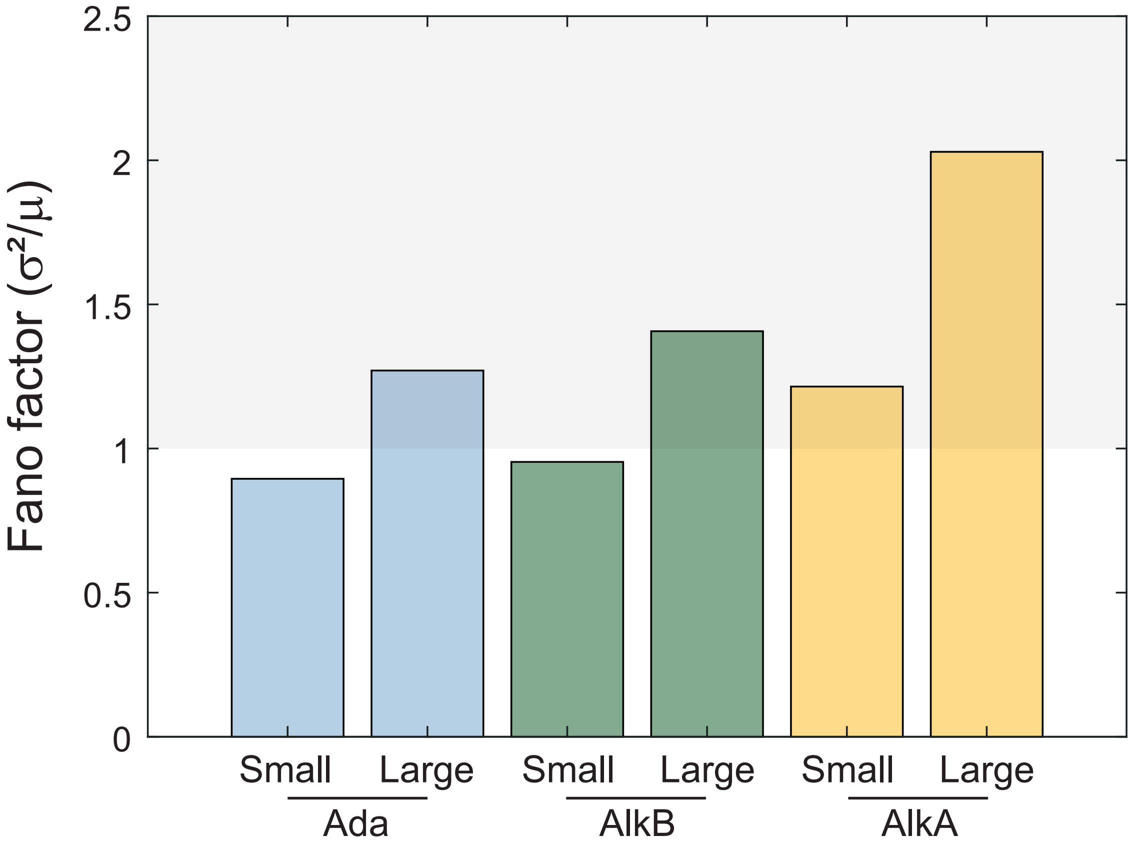

Supplementary Figure 6

Basal expression of adaptive response genes follows Poisson statistics. Fano factors (variance/mean) for the absolute copy numbers of Ada-Halo, AlkB-Halo and AlkA-Halo molecules per cell in untreated cells, using single-molecule counting data from Figure 3. Because average molecule abundance increases as cells progress in the cell cycle, we grouped cells by size with Small = cells below the mean cell length, and Large = cells above the mean cell length. Fano factors close to 1 indicate that these genes are expressed according to a Poisson process without bursting. For the larger cells variation in the cell cycle likely contributes to additional noise in expression levels beyond what is expected from a Poisson distribution.

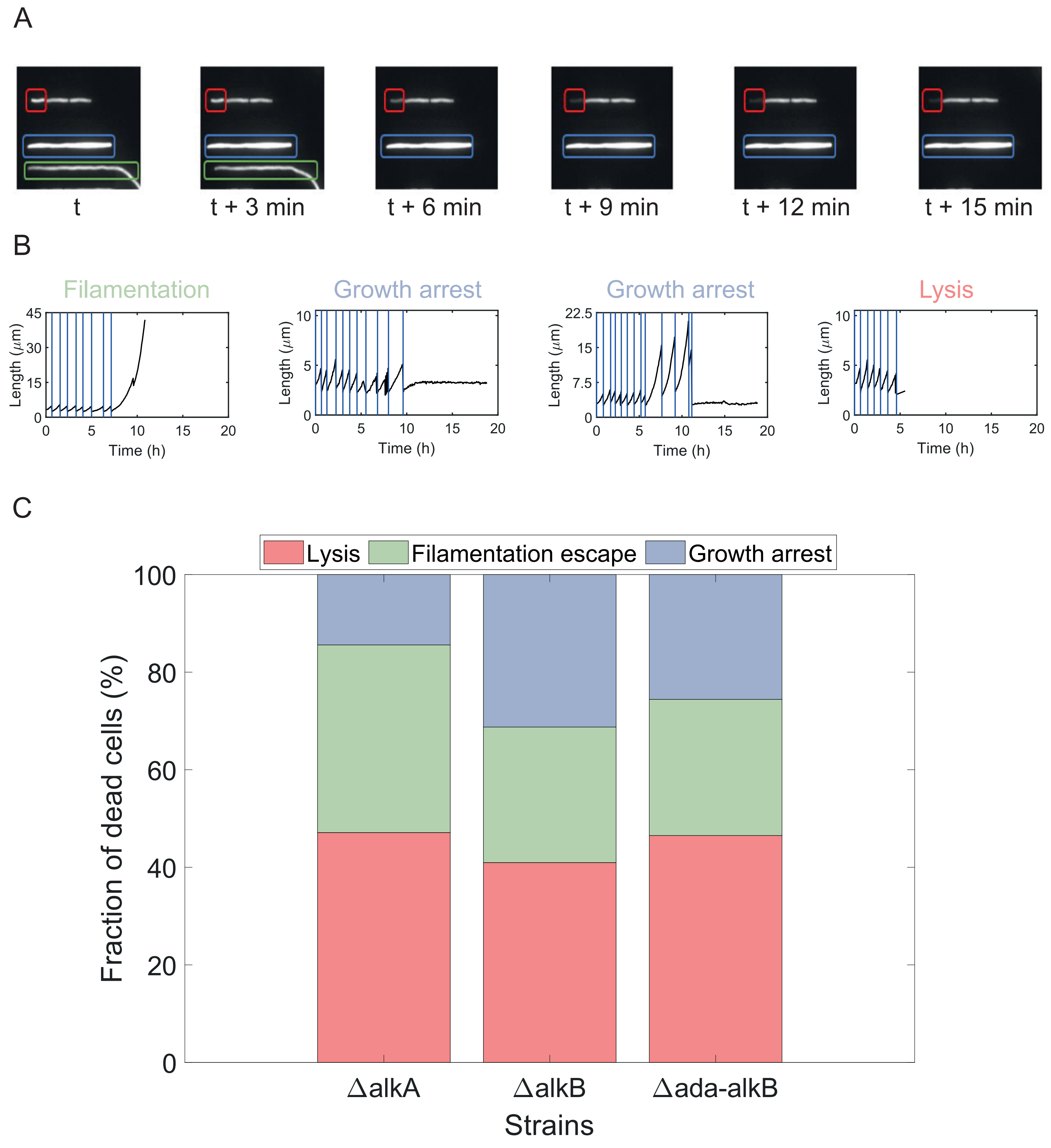

Supplementary Figure 7: Quantification of different types of cell death observed in the microfluidic experiments.

(A) Snapshots showing examples of different types of cell death with 1 mM MMS treatment: cell lysis (circled in red), cell growth arrest (circled in blue), cell escape due to filamentation (circled in green). (B) Examples of cell length time-traces showing the different types of cell death in the presence of 1 mM MMS. (C) Quantification of the fraction of lysed cells, growth arrest and filamentation for the strains *ΔalkA, ΔalkB* and *Δada-alkB* used in Figure 4.

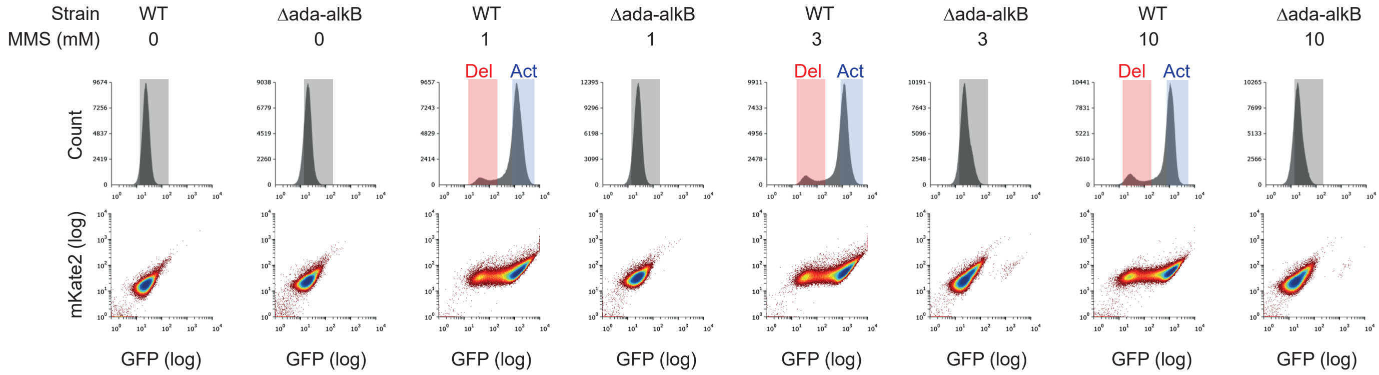

Supplementary Figure 8: Detection and sorting of Activated and Delayed subpopulations

Flow-cytometry was performed on the WT and *Δada-alkB* strains, both carrying a plasmid-based P_{ada}-*GFP* reporter and a segmentation marker mKate2. The segmentation marker enables to exclude debris and contaminants from the sorting and analysis. In absence of MMS treatment, WT cells exhibit a unimodal distribution that is used to define the population gate of the Delayed subpopulation (Del). After 90 min treatment with 1, 3 or 10 mM MMS, WT cells exhibit a bimodal distribution reflecting the subpopulations of Delayed (Del) and Activated (Act) cells. The *Δada-alkB* remains inactivated independently of the MMS concentration, confirming that the P_{ada}-*GFP* reporter is dependent on Ada.

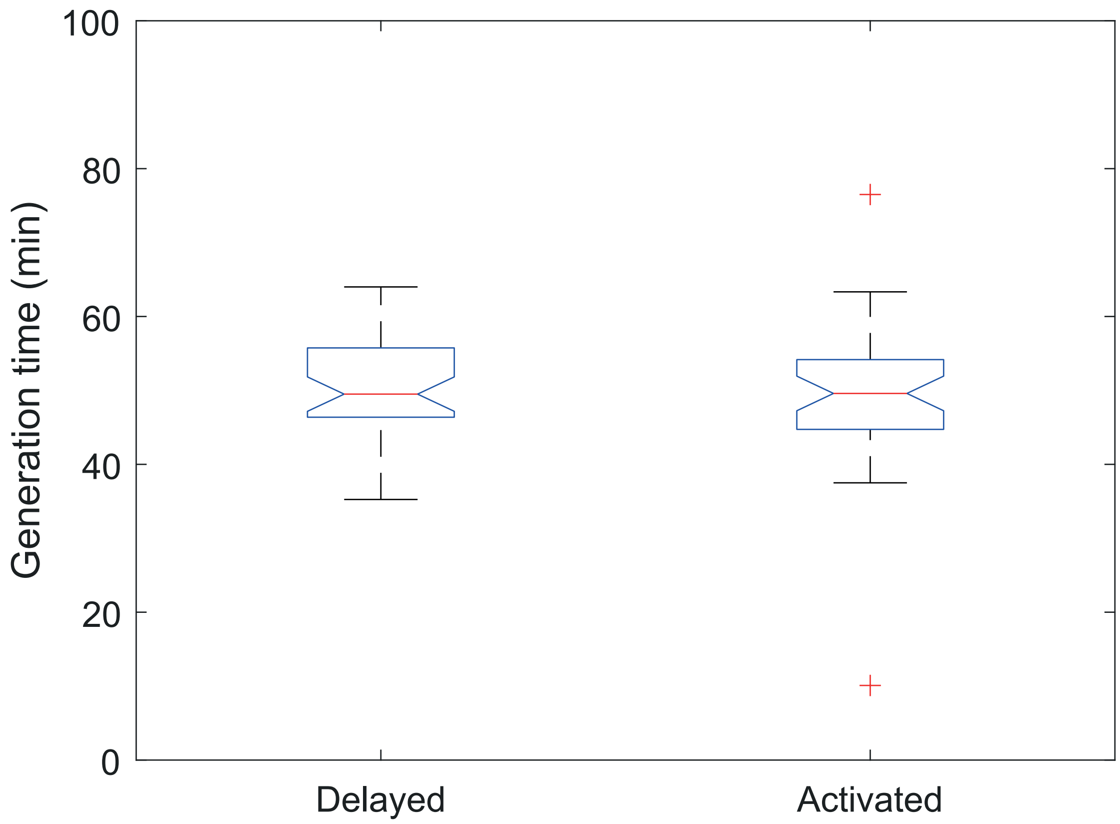

Supplementary Figure 9: Cell generation times for Delayed and Activated subpopulations.

Cells carrying a P_{ada}-*CFP* reporter were imaged in the microfluidic device with 1mM MMS treatment. Cells were defined as Delayed or Activated based on the P_{ada}-*CFP* fluorescence level and a threshold of 30 a.u.. Generation times were computed from the time interval between consecutive cell divisions, which were detected based on the mKate2 segmentation marker.

Genotype	Source	Identifier
Escherichia coli K12 AB1157	Bachmann, 1996	**OX-001**
AB1157, ΔflhD, mKate2, MutL-mYPet	Uphoff, 2018	**SU178**
AB1157, Δada-alkB::kan	This study	**SU797**
AB1157, Δada-alkB::kan, ΔflhD, mKate2, MutL-mYPet	This study	**SU852**
AB1157, ΔalkB	This study	**SU733**
AB1157, ΔalkB::kan, ΔflhD, mKate2, MutL-mYPet	This study	**SU775**
AB1157, ΔalkA	This study	**SU727**
AB1157, ΔalkA::kan, ΔflhD, mKate2, MutL-mYPet	Uphoff, 2018	**SU399**
AB1157, ada^{C321A}-alkB::cat	This study	**SU858**
AB1157, ada^{C321A}-alkB::cat, ΔflhD, mKate2, MutL-mYPet	This study	**SU902**
AB1157, ada^{C321A}-CFP::kan-alkB::cat, alkA-mYpet, ΔflhD, mKate2	This study	**SU895**
AB1157, ΔflhD, mKate2, ada-mYPet::kan	Uphoff et al., 2016	**SU072**
AB1157, ΔflhD, mKate2, alkB-mYPet::kan	This study	**SU750**
AB1157, ΔflhD, mKate2, alkA-mYPet::kan	This study	**SU749**
AB1157, ΔflhD, P_{ada}-msCFP3::kan (inserted at intS)	Uphoff et al., 2016	**SU099**
AB1157, ΔflhD, mKate2, ada-CFP::kan, alkA-mYPet	This study	**SU910**
AB1157, ΔflhD, P_{ada}-msCFP3::kan (inserted at intS), alkA-mYPet	This study	**SU753**
AB1157, ada-HaloTag::kan	This study	**SU651**
AB1157, alkA-HaloTag::kan	This study	**SU650**
AB1157, alkB-HaloTag::kan	This study	**SU647**
AB1157, mKate2, pUA139	This study	**SU828**
AB1157, mKate2, Δada-alkB, pUA139	This study	**SU829**
AB1157, ΔflhD, mKate, aidB-mYPet::Kn	This study	**SU931**
AB1157, ΔflhD, mKate2,alkB-mYPet	This study	**SU741**
AB1157, ΔflhD, mKate2,alkA-mYPet	This study	**SU739**
AB1157, ΔflhD, mKate2,ada-mYPet::kan	Uphoff et al., 2016	**SU068**
MG1655, motA-, mKate2, ada-mYPet::kan	Laboratory collection	**SU052**
MC4100, ada-mYPet::kan	Uphoff et al., 2016	**SU044**

Table S1: Strains used in this study

Reference	Sequence	Construct
SU023_ada_LambdaRed	CGCCAGTGGCTCTTGCCACGGTTCAGCATCGGCAAACAGATCCAA CATTACCTCTCCTCATAATATCCTCCTTAGTTCC	LambdaRed insertion at Ada C-ter
SU024_ada_LambdaRed	TAAAGCGCAACTGCTGCGCCGCGAAGCTGAAAATGAGGAGAGGTCGGCTGGCTCCGCTGC	LambdaRed insertion at Ada C-ter
SU025_ada_seq	ATCTGGCGAAACGGCGACTG	Sequencing of lambdaRed insertion at Ada C-ter
SU026_ada_seq	TGAAACCGTCAGTTATCAGC	Sequencing of lambdaRed insertion at Ada C-ter
SU027_alkA_LambdaRed	CCAGGCCGGATAAGGCGCTCGCACCGCATCCGGCGACCAACGAATATCCTCCTTAGTTCC	LambdaRed insertion at AlkA C-ter
SU028_alkA_LambdaRed	GTTGCATATCTGGTATACGGAAGGCTGGCAACCAGACGAAGCATCGGCTGGCTCCGCTGC	LambdaRed insertion at AlkA C-ter
SU029_alkA_seq	GGTGAGGTGATTGCCGATGC	Sequencing of lambdaRed insertion at AlkA C-ter
SU030_alkA_seq	CTTTGCGTGGCTGGCAGGCG	Sequencing of lambdaRed insertion at AlkA C-ter
SU266_Fw_AlkB_LambaRed	CCATCGACTGCCGCTACAACCTGACATTCCGTCAGGCAGGTAAAAAAGAATCGGCTGGCTCCGCTGC	LambdaRed insertion at AlkB C-ter
SU267_Rv_AlkB_LambaRed	CAGCCCGCAGTTTAAACATCTTCGCGCGCACAGCAATAATAATTCTTATTTAATATCCTCCTTAGTTCC	LambdaRed insertion at AlkB C-ter
SU268_Fw_AlkB_Seq	CGATTTTTCAATTTGGCGGCC	Sequencing of lambdaRed insertion at AlkB C-ter
SU269_Rv_AlkB_Seq	GATAAGGCGCTGATTGATAAAAGC	Sequencing of lambdaRed insertion at AlkB C-ter
SU374_Fw_Ada_A38C	AGGCATCTTTTGCCGTCCGTCTTGC	Ada A38C substitution into pMV007
SU339_Rv_Ada_A38C	GTGGTACGCACGGCGAAA	Ada A38C substitution into pMV007
SU349_Fw-del-ada-alkB	CCTGGATGTCACCACAGTTTAAAAGCTTCCTTGTCAGCGAAAAAAAATTAAGTGTAGGCTGGAGCTGCTTC	LambdaRed deletion of ada-alkB (kan selection)
SU350_Rv-del-ada-alkB	AGCCCGCAGTTTAAACATCTTCGCGCGCACAGCAATAATAATTCTTATTTCATATGAATATCCTCCTTAG	LambdaRed deletion of ada-alkB (kan selection)
SU398_Fw_pAda-Ada-AlkB	AACCTGGATGTCACCACAGTTTAAAAGCTTCCTTGTCAGCGAAAAAAAATTAAAGCGCAAGATTGTTGGTTTTTGC	Lambda Red insertion of Ada variant in ada-alkB (Cm selection)
SU399_Rv_pAda-Ada-AlkB	CACACTGATAAATGGCCAGCGATACTGCCGCCAGACAAGTACAAGAAGTTCCATCACCAGGCGTTTAAGGGCAC	Lambda Red insertion of Ada variant in ada-alkB (Cm selection)

Table S2: Primers used in this study
